# Biases in an artificial intelligence image-generator’s depictions of healthy aging and Alzheimer’s

**DOI:** 10.1093/jamia/ocaf173

**Published:** 2025-10-27

**Authors:** Channah Osinga, Natcha Jintaganon, Dirk Steijger, Marjolein De Vugt, David Neal

**Affiliations:** Department of Clinical Neuropsychology, VU University Amsterdam, Amsterdam, 1081 BT, the Netherlands; Department of Medical Informatics, Amsterdam UMC, Amsterdam, 1100 DD, the Netherlands; Department of Psychiatry and Neuropsychology, Faculty of Health, Medicine and Life Sciences, Alzheimer Centrum Limburg, Mental Health and Neuroscience Research Institute, Maastricht University, Maastricht, 6229 ET, the Netherlands; Department of Psychiatry and Neuropsychology, Faculty of Health, Medicine and Life Sciences, Alzheimer Centrum Limburg, Mental Health and Neuroscience Research Institute, Maastricht University, Maastricht, 6229 ET, the Netherlands; Department of Medical Informatics, Amsterdam UMC, Amsterdam, 1100 DD, the Netherlands

**Keywords:** artificial intelligence, ageism, dementia, bias, ethics

## Abstract

**Objective:**

This content analysis study investigates potential biases in image generation by 2 artificial intelligence (AI) tools, DALL-E 3 and Midjourney, in portraying older adults and individuals living with dementia. Despite widespread use of generative AI in various sectors, there is limited research on how these models might perpetuate stereotypes and stigmatization through their images.

**Materials and Methods:**

1056 images were generated using specified prompts categorized into 3 groups: general older adults, dementia-related, and control. Each prompt began with “photorealistic portrait” followed by specific scene descriptions. Four researchers conducted content analysis on each generated image, focusing on factors, such as portrait style, setting, posture, apparent sex of subjects, and emotional affect. The analysis was executed with blinding and randomization protocols to ensure unbiased assessment. Chi-square tests examined the relationship between prompt categories and variables.

**Results:**

Results revealed significant disparities in depictions of older adults and those with dementia compared with control images. Both models more often portrayed subjects in response to dementia-related prompts with negative affect, in less favorable emotional states. However, DALL-E 3 also generated more personas displaying positive affect in response to these prompts. Variations in depiction styles between the 2 AI models were noted, with DALL-E 3 showing a broader diversity of outputs.

**Discussion and Conclusions:**

The findings highlight AI's potential to reinforce stigmatizing stereotypes through biased image generation. Recommendations include selecting prompts carefully to avoid negative depictions and advocating for greater AI explainability and inclusivity by design. Future research should explore other AI models, other forms of bias, and strategies to mitigate biases.

## Introduction

Generative artificial intelligence (AI), particularly in the form of large language models and image generators, has garnered increased attention in the context of health and health care.^1^ Outputs of generative AI are increasingly disseminated through online media^2^ and are becoming more widely used in clinical and educational settings. Examples of such generative AI use include creation of patient education materials^3^ and the use of AI images as memory aids.^4^ As such, generative AI has the potential to significantly influence both public and professional discourse and perceptions. This raises the question of whether potential biases in underlying models might lead AI-generated media content to positively or negatively shape discourse on the important societal and public health topics.

One of the most pressing societal and public health developments of our time is the aging global population.^5^ While this demographic shift often raises concerns about burdens on society, it is crucial to recognize that older adults are active citizens entitled to good health and quality of life. However, a considerable body of research highlights how societal biases negatively impact older adults and can lead to stigma and active discrimination.^6^^,^^7^ Both negative and positive stereotypes can lead to detrimental outcomes, as societal bias around aging can obscure the rights and capabilities of older individuals. For example, older individuals might be unfairly viewed as less capable of learning new skills, leading to workplace discrimination, or they may be seen as always wise and resilient, which can result in neglecting their mental health needs. Such biases may be particularly strong with respect to public understanding and representations of chronic conditions associated with aging, such as dementia,^8^ leading to assumptions that individuals with dementia cannot communicate their needs and reinforcing social avoidance that increases isolation. These biases influence interactions and care approaches, often limiting autonomy, dignity, and access to beneficial treatments.^9^ The way in which older adults, particularly those with a condition, such as dementia, are represented in media, therefore, carries significant ethical implications.^9^ Initiatives, such as the Centre for Aging Better’s age-positive image library,^10^ have emerged to promote more diverse and empowering visual portrayals of older adults. However, the extent to which AI-generated images align with or diverge from such efforts remains underexplored.

Text-to-image models, like DALL-E and Midjourney, are trained on extensive datasets of text-image pairs scraped from the internet,^11^ which reflect societal views. Therefore, it is plausible that images generated by these models reproduce societal biases. This raises concern over a harmful positive feedback loop, wherein the images produced by generative AI models further perpetuate harmful stereotypes in the media,^12^ exacerbating societal bias. Empirical evidence supports this concern. Recent studies have revealed significant biases in leading generative AI models.^13–17^ For example, occupational biases are evident in DALL-E 2, Stable diffusion, and Midjourney, where neutral prompts frequently generate images that exclude women, ethnic minorities, and other marginalized groups.^13–15^ Representativeness bias has also been observed, as these models often omit or stereotype people with disabilities and non-normative body types, instead favoring Western beauty standards and homogenized depictions of physical appearance.^16^^,^^17^ Despite this growing literature, little research has examined how aging populations are portrayed by these models.

This study addresses this critical gap by investigating whether 2 AI-based image generators exhibit potential biases that could perpetuate or amplify stigma surrounding ageing and dementia, as an example of a chronic age-related health condition. In this study, we conceptualize bias primarily in the computational sense: as systematic patterns or deviations produced by the image generation model in response to certain prompts, reflecting how the model has learned to associate specific terms or identities with particular visual characteristics.

Additionally, the study will offer prompt recommendations for health professionals, researchers, and communications experts on how to utilize generative AI in a manner that minimizes any identified biases. This work ultimately seeks to contribute to a broader dialogue about the implications of AI in shaping public perceptions on important medical and public health topics.

## Methods

### Design

In this content analysis study, images were generated using 2 prominent AI-based image generator tools: DALL-E 3 (OpenAI) and Midjourney version 6 (Midjourney). The content of the images generated from predefined sets of prompts focused on aging and dementia was compared with content of images generated from a predefined set of control prompts. The comparison focused on individual personas depicted in the images, analyzing their affect, sex, posture, and surroundings.

### Prompt groups and structure

The prompt terms were organized into 3 comparative categories:

1. General older adult prompts
2. Prompts describing a person with dementia
3. Control prompts

Each individual prompt was divided into 2 parts: a prefix and a scene description. The prefix defined the style of the expected images, which in this study was “photorealistic portrait” for all prompts. The scene description specified the content of the image to be generated, which varied by prompt categories.

For the general older adult prompt scene descriptions, the inclusive language guidelines established by journalistic and media associations were followed, employing the term “older” as the preferred descriptor.^18^ Although the term “elderly” is regarded as less acceptable within these guidelines, it was included in this analysis for comparative purposes and due to its prevalent usage. These terms were subsequently combined with “person,” “man,” and “woman” for the development of the prompts.

For prompts describing dementia, the positive language guide was adhered to, to ensure sensitivity and alignment with best practices in representation.^19^ Two subcategories were established, reflecting major patterns in medical and colloquial terminology: “[persona] [living with] dementia” and “[persona] [living with] Alzheimer’s disease.” Scene descriptions were completed with one of 3 sex identifiers: “man,” “woman,” or “person.” This allowed assessing potential biases related to gender and neutral pronouns.

One example of a full prompt from the older adult group, therefore, reads “photorealistic portrait of an older person.” For the older adult and dementia-related prompts, all possible combinations of scene description components as described were added to the prefix to generate images for this study. Control prompts consisted only of the 3 sex identifiers (example prompt: “photorealistic portrait of a person.”). All prompts used can be found in the online Appendix.

### Image generation, blinding, and randomization

Images were generated by entering the prompts into the user interface of each AI-based tool. The generated images were downloaded and saved on the computer of the primary researcher. To ensure unbiased evaluation, the saved images were assigned file names by an independent researcher that concealed both the AI-based tool and the prompt used to generate the image. This meant that images could be analyzed blind to both model and prompt. The independent researcher maintained a key that was used to unblind the results obtained following the analysis.

**Figure 1.**
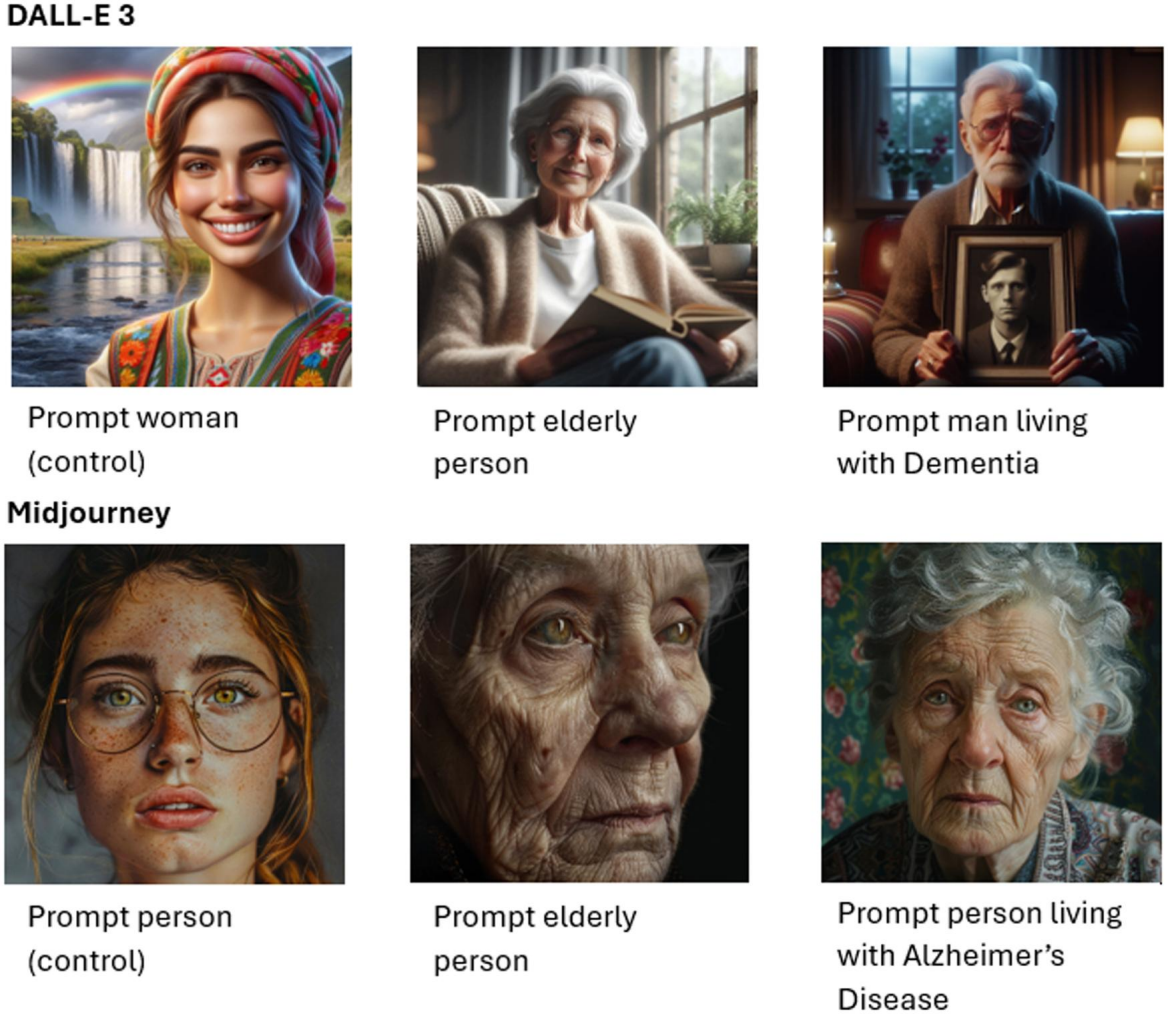
Example outputs from DALL-E 3 and Midjourney with their corresponding prompts.

A total of 528 images were generated with DALL-E 3 via the Copilot image creator website and 528 images were generated with Midjourney platform. All generated images are publicly available in the Mendeley data repository (DOI: 10.17632/5kzvssvk4d.1). Some examples of generated images are shown in Figure 1. Table 1 shows how many images were generated for each variant of the scene descriptions used. For each generative AI-based tool, a chi-square test of independence was performed to assess the relationship between the prompt categories and the portrayed subjects’ affect, setting, posture, portrait style, and apparent sex.

**Table 1.** Number of images generated by each prompt for one generative AI model.

Prompt categories	Scene descriptions	Generated images (n)
General Older Adults Prompts	Older person	28
	Older man	28
	Older woman	28
	Elderly person	28
	Elderly man	28
	Elderly woman	28
Dementia Related Prompts	Person living with dementia	16
	Person with dementia	16
	Man living with dementia	16
	Woman living with dementia	16
	Man with dementia	16
	Woman with dementia	16
	Person living with Alzheimer’s Disease	16
	Person with Alzheimer’s Disease	16
	Man living with Alzheimer’s Disease	16
	Woman living with Alzheimer’s Disease	16
	Man with Alzheimer’s Disease	16
	Woman with Alzheimer’s Disease	16
Control Prompts	Person	56
	Man	56
	Woman	56
	Total Generated Images (per model)	528

### Image analysis

Content analysis of the images was performed by 4 researchers (NJ, DN, DS, and CO), using a realist perspective, which assumes that meaning exists independently of interpretation and can be systematically analyzed. Data collection and analysis were guided by the analysis guideline that was designed for this study by DN. The guideline was piloted with an initial set of ten images to test its feasibility. One adjustment was made to provide researchers with instructions on how to record data if more than one persona was portrayed in the image, in which case the researchers determined the “primary” persona in their assessment.

In order to analyze the persona’s affect, facial expressions were categorized based on 6 cardinal emotions: enjoyment (positive affect), sadness, fear, disgust, anger, and surprise (negative affect).^20–22^ Additional options were “neutral” and “indeterminate” (neutral affect) as some images displayed no positive or negative affect or the affect could not be determined, for example, because only a part of the subject’s face was portrayed. The facial expression of each persona in the images was standardized by comparing it to an example image from the Radboud Faces Database. This database is validated and follows the Facial Action Coding System (FACS) gold standard for assessing facial expressions related to 6 cardinal emotions as well as a neutral expression.^20^^,^^23^ In theory, these expressions should be universally associated with the cardinal emotions across cultures, genders, and age. In each case, researchers were instructed to assign the closest matching expression from the example image (one of the 6 emotions or neutral). Images were further analyzed for portrait style (full length, partial body, headshot, or extreme close-up), setting (indoors, outdoors, or indeterminate), apparent sex of subjects (male, female, or indeterminate), posture (standing, semi-recumbent, sitting, lying, other, and indeterminate), and presence of assistive technology in frame (yes/no). Regarding assistive technologies, we included representations of any equipment designed to support someone with a disability, such as canes, walkers, and hearing aids.^24^

The analysis of all images was conducted independently by 4 researchers (NJ, DN, DS, and CO) with a target inter-rater reliability (IRR) of 80%.^25^^,^^26^ Any discrepancies between the researchers’ evaluations were resolved through discussion to establish a single consensus dataset for each image. To monitor consistency between the researchers’ coding decisions, IRR was evaluated after coding the first 30 images.^27^ If the 80% IRR threshold was not achieved, the sources of discrepancies were identified and addressed, followed by reassessment after coding each subsequent set of 30 images. This iterative process was repeated until the target IRR was attained. Once the IRR threshold was met, analysis continued to completion, with the final IRR calculated and reported.

### Outcomes

#### Primary outcomes

The primary outcome of this study was the difference between the 3 prompt categories in the proportion of personas in images generated by DALL-E 3 and Midjourney displaying negative, positive, or neutral affect.

#### Secondary outcomes

The secondary outcomes were differences between the 3 prompt categories in the proportion of images generated by DALL-E 3 and Midjourney coded as each portrait style and setting, and the persona’s apparent sex and posture.

Within the prompt group, statistical comparisons were beyond the scope of this study. However, data from images generated by different scene descriptions within each prompt group were inspected, and any apparent differences based on the descriptor were briefly described.

### Statistical analysis

The required sample size was based on power calculations in G*power, assuming a medium effect size, a power of 0.8, and an alpha level of 0.0125. The statistical analyses were performed using IBM SPSS Statistics for Windows, Version 26.0 (Armonk, NY: IBM Corp.). Chi-square (Chi^2^) tests were performed, or, where assumptions were not met, Fisher’s exact test was performed. The alpha level for statistical significance was set at 0.05. For the primary outcome of affect between groups, if results of the primary analysis were significant, these were followed up with pairwise Bonferroni-corrected tests comparing between each subgroup pair. Pearson’s adjusted residuals were compared with the critical value for statistical significance in order to determine which differences in the prompt groups were driving the differences.^28^ We descriptively explored within-group prompt patterns by visually inspecting the frequency distributions of the primary outcome (affect) within each prompt condition. No formal statistical tests were conducted, given the exploratory nature of these comparisons and limited power.

## Results

An IRR threshold of 80% was achieved after coding 3 sets of 30 images, with the final IRR recorded at 82%. Following piloting, no further changes were made to the data collection tool. Table 2 shows the results of the image analysis for DALL-E 3 and Midjourney and the statistical comparisons between prompt groups.

**Table 2.** Results of image analysis and statistical comparisons between prompt groups for images generated by DALL-E 3 and Midjourney.

Outcome variable	Model	Test statistic (df)	Significance	Effect size (Cramer’s V)
***Subject affect*** ^a^	DALL-E 3	X^2^ = 120.98 (4)	*P < *.001	0.34
	Midjourney	X^2^ = 17.97 (2)	*P < *.001	0.18
***Apparent Sex***	DALL-E 3	Fisher’s exact 9.75	0.06	0.10
	Midjourney	Fisher’s exact 8.02	0.05	0.10
***Portrait style***	DALL-E 3	Fisher’s exact 51.83	*P < *.001	0.21
	Midjourney	Fisher’s exact 45.05	*P < *.001	0.17
***Subject posture***	DALL-E 3	X^2^ = 73.69 (2)	*P < *.001	0.37
	Midjourney	Fisher’s exact 8.89	*P* = .002	0.15
***Setting***	DALL-E 3	X^2^ = 94.63 (4)	*P < *.001	0.30
	Midjourney	Fisher’s exact 3.91	*P* = .23	0.06

a For Midjourney, affect levels were dichotomized into “negative” and “non-negative” (neutral or positive) due to sparse representation of positive affect.

### Dall-E 3

For apparent sex and portrait style, the Chi-square test assumptions were not met. A significant difference was observed between prompt categories on subject affect, χ^2^(4, *N* = 528) = 120.98, *P < *.001. Cramer’s V was 0.34, indicating a medium effect size.^29^ There were also statistically significant differences between prompt categories on the portrait style (Fisher’s exact 51.83, *P < *.001 and Cramer’s V = 0.21), setting (χ^2^(4, *N* = 528) = 94.63, *P < *.001, and Cramer’s V = 0.30), and posture (χ^2^(2, *N* = 528) = 73.69, *P < *.001, and Cramer’s V = 0.37). None of the images contained assistive technology and all images were assigned only sitting postures or indeterminate. Therefore, the assistive technology variable and the other posture categories were excluded from the analysis.

**Figure 2.**
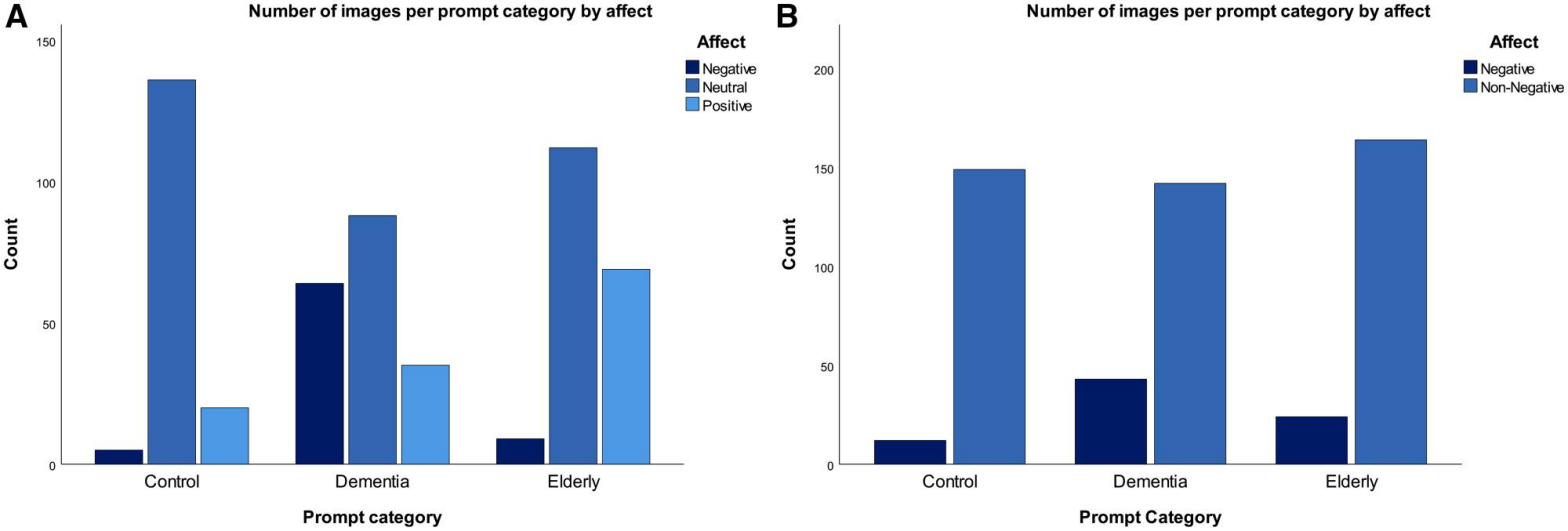
(A) Dall-E 3: bar chart showing the number of images with personas coded as expressing negative, neutral and positive affect per prompt group. (B) Midjourney: bar chart showing the number of images with personas coded as expressing negative and non-negative affect per prompt group.

Further post-hoc analysis revealed a value of 9.5 for the association between the dementia prompt group and negative affect (critical value 2.81), demonstrating that a higher proportion of images generated from dementia-related prompts depicted personas with negative affect. Pairwise comparisons with Bonferroni’s correction indicated that the proportion of negative affect was significantly higher for dementia prompts (*n *= 64, 34.2%) compared to the control prompts (*n *= 5, 3.1%) and the older adult prompts (*n *= 9, 4.7%) (Figure 2A). Additionally, a Pearson’s adjusted residual value of 5.4 was found for the association between older adult prompts and positive affect, with pairwise comparison proportions of 36.9% for the older adult prompts (*n *= 69) compared to 12.4% for the control prompts (*n *= 20) and 18.7% for the dementia prompts (*n *= 35), demonstrating that the older adult prompts resulted in more images with personas of positive affect. Moreover, indoor scenes were most common for older adult (*n *= 95) and dementia prompts (*n *= 84) and least frequent for control prompts (*n *= 12). Outdoor settings appeared more often in response to control prompts (*n *= 8) and older adult (*n *= 15) but rarely in response to dementia prompts (*n *= 1). A large number of images had indeterminate settings (dementia *n *= 102, older adult *n *= 80, and control *n *= 141). Sitting postures were most frequent in response to older adult prompts (*n *= 88), followed by dementia prompts (*n *= 59), and least in response to control prompts (*n *= 8). Indeterminate postures were frequent in all groups (control *n *= 153, dementia *n *= 128, and older adult *n *= 102). In terms of image composition, headshots were most common across all prompts (control *n *= 153, older adult *n *= 163, and dementia *n *= 142), while partial-body images were more frequent for dementia prompts (*n *= 44 vs *n *= 27 older adult, *n *= 2 control). Extreme close-ups were rare overall (≤6 per group).

### Midjourney

The Chi-square test assumptions were only met for affect and posture. Owing to the low number of positive affect images, levels of affect were collapsed into 2 groups: negative and non-negative. A significant relationship was observed between prompt categories and the collapsed subject affects, χ^2^(2, *N* = 528) = 17.973, *P < *.001. Cramer’s V was 0.183, indicating a small effect size. There were also statistically significant differences between prompt categories on portrait style (Fisher’s exact 45.05, *P < *.001, and Cramer’s V = 0.17) and posture (χ^2^(2, *N *= 528) = 8.89, *P* = .002, and Cramer’s V = 0.15). None of the images contained assistive technology and all images were assigned only sitting postures or indeterminate. Therefore, the assistive technology variable and the other posture categories were excluded from the analysis.

Pearson’s adjusted residuals indicated a value of 4.0 for the association between the dementia prompt group and negative affect. This suggests that negative affect in dementia prompts occur significantly more often than expected, constituting 23.2% of images from the dementia prompt group (*n* = 43), compared to 7.5% for controls (*n* = 12) and 12.8% for the older adult group (*n* = 24) (Figure 2B). For posture, sitting figures were present only in the dementia prompt group (*n* = 6), with none in response to older adult or control prompts. The majority of postures were indeterminate across all groups (*n* = 161 control, *n* = 179 dementia, and *n* = 188 older adult). Moreover, extreme close-ups appeared only in the control group (*n* = 12) and were absent in both the dementia and older adult prompt groups.

### Within prompt group comparisons

Inspection of data from images generated by different scene descriptions within each prompt group suggested distinct affective patterns influenced by the phrasing of the prompts. DALL-E 3’s output from prompts in the dementia group including “with dementia” seemed more strongly associated with negative affect than “living with dementia” or “with Alzheimer’s Disease.” The prompt “living with Alzheimer’s Disease” seemed less associated with negative affect and generated a similar number of images classified as negative affect as the older adult and control prompts, although it was less associated with positive affect personas. There were more positive affect personas coded in response to prompts “with Alzheimer’s Disease” in the dementia group and “elderly” in the older adult group. In the Midjourney images, dementia-related prompts, including “living with Alzheimer’s Disease,” “with Alzheimer’s Disease,” and “with dementia” seemed more associated with negative affect of personas.

## Discussion

This study aimed to investigate whether AI-based image generators exhibit biases that could perpetuate or amplify stigma surrounding ageing and dementia. Additionally, the aim of this study was to provide recommendations for health professionals, researchers, and communication experts on how to utilize generative AI in a manner that minimizes any identified biases. This work ultimately seeks to contribute to a broader dialogue about the implications of AI in shaping public perceptions on important societal and health topics.

Results showed that DALL-E 3 and Midjourney produced significantly more images of personas considered to exhibit negative affect in response to dementia-related prompts, compared to prompts concerning general older adults or control prompts. The effect size was larger between prompt groups in DALL-E 3 than Midjourney. Responding to prompts related to older adults, DALL-E 3 was more likely to generate images with personas displaying positive affect, compared to control and dementia-related prompts. Moreover, both tools showed a pattern of depicting personas sitting in response to dementia-related prompts. For DALL-E 3, both older adult and dementia-related prompts resulted in more personas in sitting postures compared to control prompts, while for Midjourney, this effect was specific to dementia-related prompts. Furthermore, DALL-E 3 portrayed proportionally more personas in indoor settings when responding to dementia-related prompts. Comparing between scene descriptions within prompt groups, the descriptor “with dementia” seemed to be more strongly associated with negative affect personas than other descriptors in both tools.

DALL-E 3 and Midjourney seem to exhibit particular biases responding to dementia-related prompts, and to a lesser extent, prompts related to older adults. These biases likely stem from the datasets used to train the underlying models of these AI-based tools. Both tools were trained on publicly available images and those images’ associated captions.^12^^,^^30^ Given that previous research has highlighted pervasive negative stereotypes concerning both healthy aging and people living with dementia in the media and public discourse,^6^^,^^8^^,^^9^ it is not surprising that the tools seem to manifest similar biases. For example, the bias in Midjourney that depict personas in seated postures when responding to dementia-related prompts may reinforce perceptions of aging and dementia as passive or limiting conditions that restrict physical and social activity.^31^ While some individuals may experience these feelings and phenomena as they age at various times,^32^ this should not define the condition as a whole. Many people with dementia continue to maintain their independence and autonomy through supported decision-making, such as choosing their daily activities or managing their routines with the help of technology or caregivers.^33^ Interestingly, DALL-E 3 showed signs of positive bias towards aging. This model was more likely to depict older adults with positive affect, which aligns with research showing that cultural perceptions of aging can vary widely. While aging is often associated with decline and loss, it can also be linked to wisdom and contentment.^34^ Different cultural biases in the training data for each model might explain this positive bias. However, little information about training methods and fine-tuning is available for either Midjourney or DALL-E 3. Open AI (the developer of DALL-E 3) claims to have strategies to prevent all biases in general.^35^ While we did not conduct wider comparisons between the models, anecdotally, the outputs of DALL-E 3 seemed to constitute a more diverse set of images compared to Midjourney across several variables in this study, such as affect, setting, and posture, which may reflect the impact of such mitigation efforts. However, we are not able to determine whether bias-reducing strategies could result in an “over-correction” leading to positive bias. Notably, positive stereotypes can also be damaging to public discourse if uncritically promulgated,^36^ highlighting the importance of considering this result in future research and practice.

Furthermore, the study found that the prompt “living with dementia” resulted in more positive depictions compared to “with dementia” or “with Alzheimer’s Disease.” By emphasizing the individual rather than the condition, “living with dementia” aligns with a more humanizing perspective. For the Alzheimer’s prompt, one possible explanation might be that “Alzheimer’s” may be perceived as a more clinical or scientific term within AI training data compared to dementia, leading to more neutral representations. These results reinforce the importance of carefully chosen terminology in both AI prompts and real-world discourse. In addition to previously documented biases in generative AI models,^13–17^ our findings show potential additional representatives biases related to ageing and dementia. This contribution broadens the scope of existing research on biases in generative AI and underscores the need for more inclusive model evaluation frameworks that account for age-related and cognitive diversity.

Based on this study’s findings, we can make several recommendations to users of AI-based image generators about prompts to minimize risk of generating stereotyped and potentially stigmatizing images. First, describing an older adult is preferable to references to dementia or Alzheimer’s Disease, and most important to avoid is the terminology “[man/woman/person] with dementia.” Second, the prompt writer should consider specifying the emotional affect and posture of the persona and the setting in which the portrait should be placed, based on a conscious choice regarding what they want to portray, to which audience, and why. Third, if the above-mentioned features of the image are not specified, users should reflect critically on generated images with these biases in mind and should consider revising the prompt or attempting to generate a new image with the same prompt if they note unwanted biases. Users should bear in mind that Midjourney may require the user to provide more diverse, detailed, or nuanced prompts as input, in order to generate varied outputs, whereas users should expect more diverse possible responses to a single prompt from DALL-E 3.

Beyond prompt design, we also recommend prioritizing broader education and guidance about potential biases. This includes developing and providing accessible resources, such as tutorials, workshops, and user guides that inform users about AI biases, ethical considerations, and best practices. Integrating these educational tools directly into AI platforms may empower users to critically assess generated content and make more informed decision. Finally, it should be noted that there are alternatives to using AI image generators at all, in the form of image libraries, such as that maintained by the Centre for Aging Better in the UK,^10^ which is curated to provide more balanced, respectful portrayals of older adults. This remains an important approach to avoid reinforcing harmful stereotypes.

One of this study’s strengths lies in the objective approach employed during the evaluation process. The researchers were blinded to the categorical properties of the images during the evaluation process, which minimized potential bias. Furthermore, while different cultural backgrounds of the researchers could have influenced interpretations of affect, this study achieved an inter-rater reliability (IRR) exceeding 80% after 3 rounds of coding and discussion. This suggests a high level of cross-cultural agreement, reinforcing the robustness of the findings. However, some limitations should be considered. First, a key limitation of our study is that we did not directly compare the frequency or distribution of visual characteristics in generated images to real-world population data. While our analysis revealed patterns in how aging and dementia are represented by generative models, we stop short of making claims about how representative these images are of actual demographics or experiences. Moreover, the generalizability of these findings may be constrained by the specific prompts and tools used in this study as different generative AI tools or prompt designs may yield different results. We made an effort to minimize this limitation by carefully selecting widely used AI-tools and employing standardized prompts to ensure consistency in the evaluation process. We explicitly chose to use simple, minimally specified prompts (eg, “elderly man” and “person with dementia”) in order to isolate the representational impact of aging and dementia terms themselves, without interference from more descriptive or directive scene elements. However, we acknowledge that additional attributes, such as clothing, brightness, sharpness, gesture, and the presence of objects or companions may also influence the portrayal and perception of ageing. For instance, the inclusion of caregiving roles in prompts could add a crucial social dimension to these portrayals. Another limitation is that the current study focused exclusively on AI-generated photorealistic portraits to enable a controlled and systematic analysis of visual biases. Portrait-style images provide a consistent format for comparison and reduce variability introduced by complex backgrounds or contextual elements. However, this approach limits our understanding of how AI models represent individuals in more naturalistic, everyday contexts. Finally, our study relied solely on visual analysis, which may not fully capture the complexity of the generated images.

Future research should consider expanding the prompt space to include, for example, social relationships and scenes of daily living, to better capture the complexity of ageing representation. Second, analyzing the diversity of outputs produced by generative AI tools using different quantitative measures, such as entropy or the Gini index,^37^^,^^38^ could offer deeper insights into the observed differences and provide suggestions for helping to refine AI-based image generator tools for more balanced and diverse output generation. Third, extending this line of research to other popular generative AI tools, such as Stable Diffusion, Gemini, and other emerging tools, would provide a broader understanding of how different AI-based image generator tools depict aging and dementia. Prior evidence^39^ suggests that AI generated images of mental health conditions rather reflect cultural stereotypes rather than evidence-based clinical realities. Our findings on dementia support this pattern and highlight the importance to examine whether similar distortions appear in AI depictions of other common mental health conditions like depression, anxiety disorders, or schizophrenia. Crucially, future work should move beyond merely documenting such biases and actively evaluate interventions designed to mitigate bias. For instance, the framework proposed by Mehrabi et al.^40^^,^^41^ identifies intervention points across the data-to-algorithm, algorithm-to-use, and user-to-data phases and may provide a useful foundation for technical solutions, governance strategies, and participatory design practices.

In conclusion, this study reveals potential biases in how 2 popular generative AI tools DALL-E 3 and Midjourney represent older adults and people living with dementia, raising critical ethical and policy considerations. Being aware of these potential biases is essential to understanding their impact and ensuring that generative AI systems are used responsibly in representing diverse populations. For users, this study offers practical strategies to minimize bias. For researchers, the study provides a foundation for extending this analysis with other prompts and to other generative AI tools, advancing understanding of how these technologies interact with sensitive social issues, and promoting ethical AI development.

## Supplementary Material

ocaf173_Supplementary_Data

## Data Availability

The data underlying this article are available in Mendeley Data at https://data.mendeley.com/datasets/5kzvssvk4d/1, and can be accessed with doi: 10.17632/5kzvssvk4d.1.
